# Exploring the role of the Recovery College model as a transformative tool for recovery-oriented practice: perceived benefits and perspectives from health practitioners in Quebec, Canada

**DOI:** 10.3389/fpsyt.2024.1440840

**Published:** 2024-09-03

**Authors:** Julie Bellemare, Catherine Vallée, Catherine Briand, Anick Sauvageau, Marie-Josée Drolet

**Affiliations:** ^1^ Department of Occupational Therapy, Université du Québec à Trois-Rivières, Trois-Rivières, QC, Canada; ^2^ Research Center, Institut universitaire en santé mentale de Montréal, Montréal, QC, Canada; ^3^ School of Rehabilitation, Faculty of Medicine, Université Laval, Québec, QC, Canada

**Keywords:** Recovery College, recovery, training program, occupational therapy, continuing professional development

## Abstract

**Introduction:**

Mental health practitioners (MHPs), including occupational therapists (OTs), need support to adopt a truly recovery-oriented practice. Like other practitioners, if OTs often embrace the principles of recovery as a philosophical foundation for their practice, these principles may not always reflect in their attitudes, behaviors or in their interventions. While further research is needed to demonstrate the positive effects of recovery-oriented training programs on MHPs’ attitudes and practice, there is a need to explore novel training programs. The Recovery College (RC) model is one of the interventions that are designed to facilitate these changes in practice, through co-production and co-delivery of recovery-focused courses curriculum. Although the perceived benefits and outcomes of RC courses are widely documented, very few studies focus specifically on what MHPs gain from them or on their global experience. The aim of this article is to describe the experience of MHPs learners in RC courses and the perceived benefits on their practice.

**Methods:**

An exploratory descriptive qualitative study was conducted. Data were collected through semi-structured interviews and analyzed using Miles and Huberman’s stepwise qualitative analysis method.

**Results:**

Participants were 13 MHPs working in community organizations or healthcare institutions and who participated as learners in a RC, in the province of Quebec, Canada. Ten themes emerged from the qualitative analysis. Participants expressed their perspectives on the format of the courses, their initial expectations and their recommendations. They also identified the types of knowledge they shared during the courses. Participants reported changes in their practice, raised awareness on their clinical and personal issues, improved well-being and recovery. Group composition, interactions within the group, complementarity of the different types of knowledge, and pedagogical design and learning activities were identified as key ingredients of RC.

**Conclusions:**

This study highlighted RCs’ role in enriching MHPs clinically and personally. RC curriculum and courses drive changes in practice and attitudes towards service users. RCs may assist MHPs reflect on practice and improve their clinical reasoning. This study advances understanding of a promising, accessible training program for adopting a recovery-oriented practice amid a paradigm shift among MHPs and OTs.

## 1 Introduction

Over the last two decades, mental health practitioners had to navigate through a paradigm shift in mental health, as evidenced by the policies and mental health action plans in many countries, such as Canada (1, 2), the United States of America (3), England (4), New Zealand (5, 6) and Australia (7). Traditionally, healthcare systems have focused on reducing and eliminating problems, deficits, and dysfunction. Healthcare systems embracing the recovery paradigm support individuals in rebuilding and redefining their lives, transcending residual symptoms of mental disorders (8, 9). Mental health practitioners (MHPs), including occupational therapists (OTs), must now integrate the values, principles, attitudes, and behaviors that are closely associated with the recovery paradigm.

Recovery-oriented practice is the application of a set of competencies that supports individuals to recognize and take responsibility for their own recovery and well-being, and to become empowered and self-determined in their own lives (10–12). A recovery-oriented practice integrates principles such as self-determination and individualized care, and emphasizes hope, social integration, community involvement, personal goal setting, and self-management (13). Recovery-oriented practice requires a redesign of the practitioner-service user relationship by sharing power equitably with service users, with the role of the mental health practitioner is to be “on tap not on top” (14).

The ongoing paradigm shift within the healthcare system remains in progress. In a recent report, the World Health Organization (15) outlined key actions for transforming healthcare systems. These include bolstering promotion and prevention efforts and establishing accessible, affordable, and high- quality health services rooted in local communities. To advance healthcare transformation, intersectoral actions are necessary at system, organizational, and individual levels (16–18). Within this context, Perkins and colleagues highlighted the crucial role of frontline workers in driving meaningful changes “from the bottom-up” (9).

Occupational therapists are well positioned to serve as primary catalysts for the advancement of recovery-oriented practice for several reasons. First, the philosophical foundations and core values of the profession are well aligned with those of the recovery paradigm (19–22). Second, OTs are expected to analyze all the factors that may explain what facilitates or impedes occupational and social participation, including exploring existing potential opportunities. They are trained to reduce environmental and societal barriers and to facilitate engagement in a repertoire of meaningful and desired occupations (21, 23). Lastly, OTs assist service users in restoring hope and a meaningful life, in (re-) building a positive identity, and in reclaiming control over their life (21). Hence, OTs are already embracing some of the critical elements of recovery-based practice, although further work is needed in terms of advocating for system change (21).

## 2 Background

Many changes are needed to implement a true recovery-oriented practice. Some of these changes relate to the clinical processes and actions performed by MHPs (the what), while others are more subtle such as the manner, attitudes and rationale underlying interventions (how and why) (24–26). For example, within clinical processes, recovery-oriented assessments diverge from the traditional goal of identifying illness and planning treatment. Instead, they focus on developing and validating personal meaning, reinforcing strengths, promoting personal responsibility, supporting a positive identity, and cultivating hope (24). A systematic review indicates that MHPs commonly utilize assessment tools focused on limitations and impairments, thus constraining a strength-based perspective that emphasizes the individual’s strengths and resources (27). This perpetuates a dichotomous perspective on health, where one is either healthy or ill, inconsistent with the recovery paradigm (28, 29).

Recovery-oriented practice is also influenced by the values, policies, and procedures of healthcare institutions, which may or may not be conducive to recovery. MHPs reported struggling to make sense of recovery-oriented practice due to competing priorities at different levels of the healthcare system (30). An increasing number of publications and initiatives on recovery-oriented practice actively involve occupational therapists, such as peer interventions (31, 32) and support for self-determination (33), suggesting their contribution in organizational transformation and paradigm shift (19, 21).

Some of the needed changes are more subtle and nuanced, such as the need for practitioners to introduce critical reflexivity on their own biases and assumptions. Unfortunately the presence of stigmatizing attitudes and behaviors amongst MHPs, known to have detrimental effects on therapeutic processes and health outcomes, is still frequently reported (34–36). In spite of explicit professional guidelines inviting OTs to advocate for service users and take actions against social and occupational injustices (10, 37), there is growing evidence that OTs are not immune to implicit biases and contribute at times to stigmatization, such as ableism, ageism, or racism (38, 39). This type of stigmatization may arise involuntarily and below the level of conscious awareness (40). Developing the professional competency to recognize this form of stigmatization within oneself is essential for minimizing personal bias and inequitable behavior rooted in social position and power (37).

Recovery-oriented training programs remain a key approach to developing recovery-oriented practice within the healthcare system (41, 42). To the best of the author’s knowledge, there is no operational definition of what a recovery-oriented training program is, and what is identified as a recovery-oriented training program varies widely in subject matter, format, length, design, and provider (43, 44). Perkins and colleagues (9) advocate for recovery-oriented training programs that include the following features: (a) content that goes beyond recovery principles and addresses recovery-oriented interventions; (b) teaching methods that involve exploring and utilizing learners’ ideas, competencies, and experiences; and (c) learner’s groups composed of individuals with diverse expertise and backgrounds/professions.

The effectiveness of recovery-oriented training programs in fostering changes in knowledge, attitudes, and interventions among MHPs need to be supported by further evidence. Two literature reviews (43, 44) suggest that while these programs have shown a moderate impact on practitioners’ knowledge and attitudes (excluding stigmatizing attitudes), they do not significantly influence practices. However, caution is advised in drawing definitive conclusions due to significant methodological heterogeneity among the studies included in these reviews, which varied in training formats, assessment methods, and study designs. Moreover, it is noted that the recovery-oriented training programs evaluated in these reviews do not align with all the features identified by Perkins and colleagues (9).

Recovery Colleges (RCs) emerged as promising entities for transforming the healthcare system that embody the values and principles of the recovery paradigm across their governance and courses curriculum (45–48). They are at the intersection of health promotion approaches, including primary, secondary, and tertiary prevention, and personal recovery (48). RCs are learning centers directly accessible to anyone interested in mental health and recovery, including those with experiential knowledge gained through life experience with mental health as a service user or relative, clinical knowledge gained and applied in the field as a mental health practitioner, and theoretical knowledge gained through academic, college, or university training in mental health.

The RC model is characterized by: (a) the creation of a co-learning space that recognizes the differences and value of each individual; (b) the sharing and recognition of different forms of knowledge; (c) the cross-fertilization of knowledge; (d) the mixing and hybridization of learners from different backgrounds (48–51). There is a correspondence between these characteristics and those that should shape a recovery-oriented training program as defined by Perkins and colleagues (9).

The current status of RCs worldwide shows that these centers are present in 28 countries across 5 continents (52). Many RCs reported the engagement of health practitioners in co-producing and co-facilitating courses (53). Hayes and colleagues (54) found that among the 59 RC surveyed, OTs were the most prevalent category of practitioners involved in the coproduction and cofacilitation of RC courses (41% of reported involvement).

In the last decade, approximately eighty studies (including 59 with evaluation data) have documented the key and active ingredients, implementation experiences, cost-effectiveness, outcomes and perceived benefits of RC courses (55, 56). For MHPs, attending a RC offers a valuable opportunity for reflective practice and some continuing professional development activities at a lower cost (57). While the outcomes and perceived benefits of RCs are increasingly documented, few studies specifically investigate what MHPs gain from participating in a RC as learners (58, 59). According to Perkins and colleagues (60), evidence regarding MHPs as learners is anecdotal. To our knowledge, no study specifically focuses on this category of learners who attend a RC, which will now be identified as mental health practitioners learners (MPHs learners).

The aim of this article is to describe the experience of MHPs learners, including OTs, in RC courses and the perceived benefits on their practice.

The three research questions are:

1. How do MHPs describe their experience as learners in RC courses?
2. What are the perceived benefits of the RC courses on their practice?
3. Based on their experience, what do MHPs learners identify as key and active ingredients of the RC model?

## 3 Methods

### 3.1 Design

An exploratory descriptive qualitative design was utilized due to the limited existing research on the perceived benefits of RC courses on MHPs learners. This design is valuable for understanding a phenomenon from the participant’s perspective (61, 62).

This study is part of a larger evaluative research project funded by the Canadian Institutes of Health Research aimed at documenting outcomes and perceived benefits of RC courses among different categories of learners (63–65). This study addresses the questions posed by the larger evaluative research project but focuses specifically on MHPs learners.

### 3.2 Study setting

The study was conducted at the Health and Recovery Learning Center-Centre d’Apprentissage Santé et Rétablissement (CASR), the RC of the province of Quebec, Canada.

Established by an OT professor and researcher in 2019, the CASR is the only French-language RC in Canada. In the fall of 2020, in response to the Covid-19 pandemic, CASR adapted all its courses to online short-format in order to quickly reach as many people as possible. At the time of data collection for this study, the CASR provided only an RC curriculum and online courses for the French-speaking population of the province of Quebec (three sessions of two hours each, totaling six hours). Currently, it has expanded its offerings to include face-to-face courses in French and online courses in Italian.

CASR governance is multi-partner and multi-sectoral (including health, education, community, civic, and research), meaning that roles and responsibilities are shared between several partners from different sectors. Partner organizations are invited to submit ideas for topics of relevance to their members, in order to contribute to CASR’s courses offerings. Several topics were covered, such as youth mental health, recovery, mental disorders, well-being and mental health, social networks and support, workplace mental health, social inclusion and living better together. Many OTs and OT students actively participate in CASR activities. They are founding members, trainers, partner organization members and learners.

The distinctive features of these courses encompass the following: (a) they are co-developed and co-delivered by a dyad of trainers (peer and practitioner trainers); (b) they employ innovative, participative, and active pedagogical methods; (c) they foster the hybridization and cross-pollination of theoretical, clinical and experiential knowledge. The courses are crafted as interactive workshops, incorporating a blend of reflection, discussion and co-production activities, experiential testimonials, and theoretical content.

### 3.3 Sampling and recruitment

The sampling and recruitment processes for this study were integrated into the data collection procedures for the larger evaluative research project. During the 2020-2021 academic year, all learners who have taken a course at CASR have been invited to take part in the research (n=379 learners). Participation in the larger project entailed completing questionnaires and sitting through an individual interview for 60 to 90 min. A total of 27 interviews were conducted between 2020 and 2021, with a diverse sample of learners (i.e.: university students, citizens, peer support workers, education professionals, health practitioners, health managers).

Out of these, eight interviews were retained as participants met the specified inclusion criteria for the current study. These criteria include: (a) being a mental health practitioner directly engaged with mental health service users or their relatives, or providing clinical support to other practitioners; (b) working in the healthcare sector (public, private, or community-based), and (c) having completed at least 2 out of 3 sessions of at least one course from the CASR.

**Figure 1:**
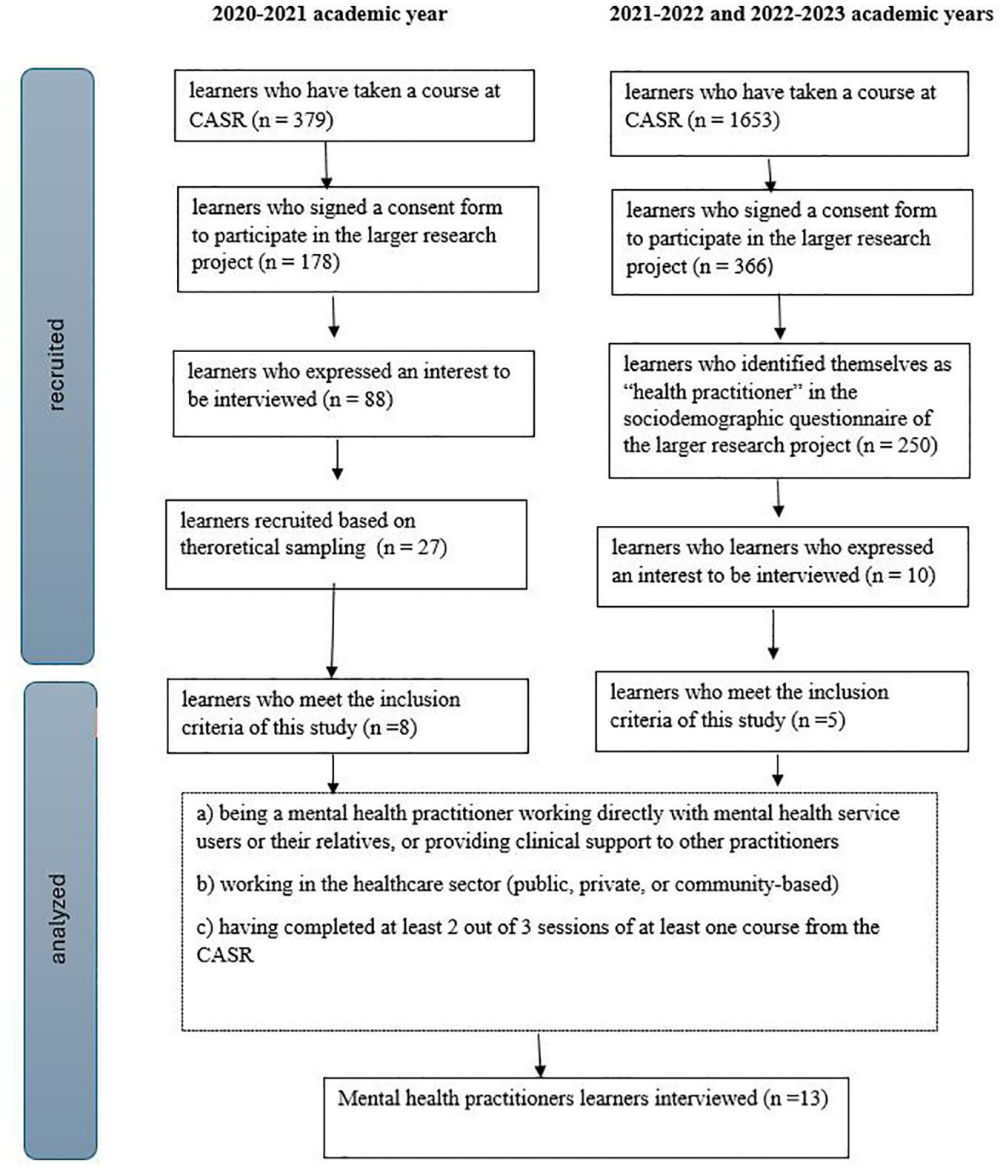
Flowchart of the sampling and recruitment of the study.

Following the analysis of these eight initial interviews, and to ensure data saturation was reached (66), a new wave of recruitment was conducted during the academic years 2021-2022 and 2022-2023. This recruitment was pursued with learners who self-identified as health practitioners in the sociodemographic questionnaire of the larger evaluative research project (n=250). Ten MPHs learners expressed interest to be interviewed, and five of them met the inclusion criteria, resulting in a total of 13 interviews for this sub-study. Further details regarding the sampling and recruitment processes are illustrated in **Figure 1**.

### 3.4 Data collection

Four members of the research team conducted semi-structured interviews on videoconference platforms (Zoom and Microsoft Teams): two doctoral students, authors of this article (JB, AS), and two research professionals (JMM, MB). All interviewers are members of the research team. These research team members are under the supervision of the principal investigator. The principal investigator (CB) plays the role of liaison, keeping the CASR and partner organizations informed of the progress of the research project. Operations of the CASR, who hosts the RC, are overseen by CASR staff and partner organizations, at arm’s length from research team.

All interviews were recorded on cloud-based video conferencing platforms, automatically transcribed, and then manually adjusted to rectify grammatical and content-related errors. Participants’ names were anonymized and replaced by unique study identifiers to ensure confidentiality. The other members of the research team or the CASR never accessed the master list.

An interview guide was designed for the data collection of the larger evaluative research project which encompassed three thematic areas: (a) expectations, objectives, and experiences at the CASR; (b) understanding of the RC model and key and active ingredients (mechanisms of action); and (c) perceived benefits. For the purposes of this study, the interview guide was enriched by the addition of four questions specifically aimed at MHPs learners. These questions concern the perceived benefits in their clinical practice and their links with key and active ingredients (e.g., I would like to know what this type of learning has brought you. Can you give me some concrete examples related to your clinical practice?). Five additional questions were also added, focusing on various aspects of clinical practice: attitudes and behaviors, relationships with service users, choice of assessments and interventions, and development of professional competencies (e.g., Have you made any changes to your behavior and attitudes in your interactions with service users? If so, what specific changes have you made?).

### 3.5 Data analysis

The data were analyzed with NVivo 14. The stepwise qualitative analysis method developed by Miles and Huberman (67) was used. The stepwise qualitative analysis method consists of three stages of activities: (a) coding data, (b) grouping into themes; and (c) validating themes.

A codebook with themes and definitions arising from the analysis was created through an iterative process by the first author of this article (JB). To ensure the rigor of data analysis, a 30% counter-coding of the interviews was conducted by the principal researcher of the larger evaluative research project (CB). To ensure consistency, intercoder reliability was calculated using the method proposed by Miles and Huberman (68) by dividing the number of agreements by the total sum of agreements and disagreements. These authors recommend a standard of 80% agreement on 95% of codes as a benchmark. The average inter-coder agreement between the two coders stood initially at 78%. Coding discrepancies were resolved through discussions between the two coders (JB, CB) until a consensus was reached. Subsequently, the lead coder (JB) adjusted the overall coding based on this consensus. Two research coordinators (LC, JNT) validated the codebook, encompassing definitions and quotes. This counter-analysis step ensured the exclusivity of themes and categories, accurate representation of quotes within definitions, and correct classification of each quote into its respective theme and category. This process allowed for final adjustments to be made.

### 3.6 Ethical considerations

The larger evaluative research project was approved by the research ethics committees of the Université du Québec à Trois-Rivières (#CER-20-270-07.01) and the Centre intégré universitaire de services sociaux et de santé de l’Est-de-l’Ile de Montréal (#MP-12-2021-2421). Each MPHs learners signed an informed consent document, after an information session, before entering the study. This informed consent was validated again at the start of each interview. All the data was securely stored on servers accessible exclusively to members of the research team.

## 4 Results

The study results will be divided into four sections. The first one will detail the sample, while the following ones describe the results associated with each research questions.

### 4.1 Sample description

Thirteen participants took part in the study. The majority were women (n=10), with an average age of 43 years, ranging from 28 to 53. The highest level of education varied from college to doctoral degree, with the majority of participants having a master’s degree (n=7). The participants reported holding at least two types of knowledge (theoretical and clinical knowledge). Six participants also reported having experiential knowledge. Six different job titles were listed by participants, psychosocial practitioners (n=3) and OTs being the most common (n=3). Most of the participants worked in healthcare institutions (n=10), while three of them were employed in community organizations. Participants’ profiles are described in **Table 1**.

**Table 1:** Description of the participant sample.

Participant	Gender	Age (years)	Highest Level of Education	Mental Health Knowledge^1^	Job Title	Workplace
**Participant 1**	F	41	master’s degree	clinical, theoretical, experiential	psychosocial practitioner	community organization
**Participant 2**	F	43	bachelor’s degree	clinical, theoretical	social worker	healthcare institution
**Participant 3**	F	35	college	clinical, theoretical	psychosocial practitioner	community organization
**Participant 4**	F	47	master’s degree	clinical, theoretical, experiential	occupational therapist	healthcare institution
**Participant 5**	F	48	master’s degree	clinical, theoretical, experiential	nurse	healthcare institution
**Participant 6**	F	51	master’s degree	clinical, theoretical, experiential	psychoeducator	healthcare institution
**Participant 7**	M	47	bachelor’s degree	clinical, theoretical	social worker	healthcare institution
**Participant 8**	M	35	master’s degree	clinical, theoretical, experiential	psychosocial practitioner	healthcare institution
**Participant 9**	F	47	bachelor’s degree	clinical, theoretical	psychoeducator	healthcare institution
**Participant 10**	F	34	master’s degree	clinical, theoretical	occupational therapist	healthcare institution
**Participant 11**	F	53	doctoral’s degree	clinical, theoretical, experiential	psychologist	healthcare institution
**Participant 12**	F	50	bachelor’s degree	clinical, theoretical	occupational therapist	healthcare institution
**Participant 13**	M	28	master’s degree	clinical, theoretical	psychosocial practitioner	community organization

^1^More than one type of mental health knowledge is possible per participant.

**Figure 2:**
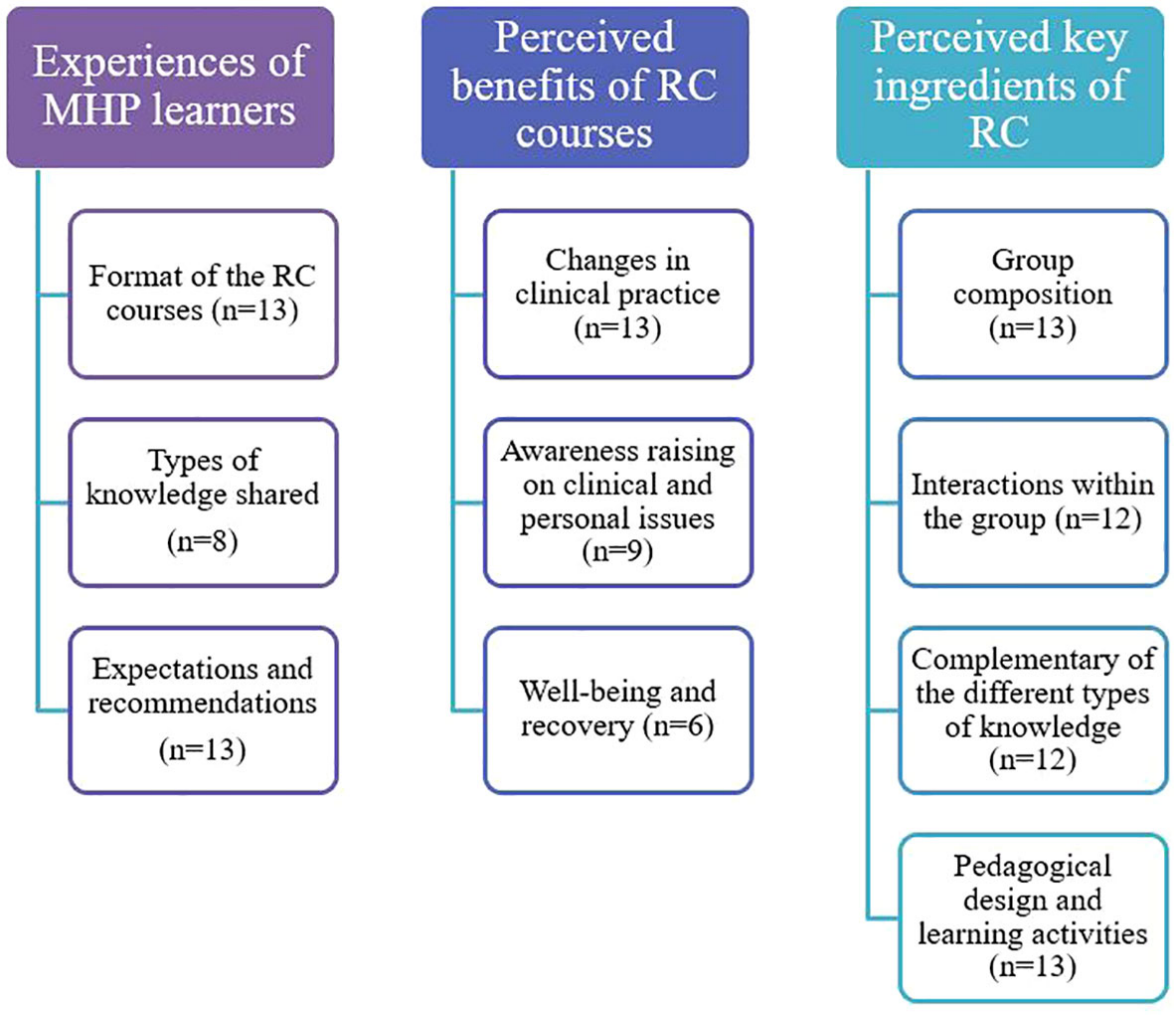
Synthesis of the themes associated with each of the three research questions.

To ensure transparency, the number of participants (n) is specified for each element emerging from the qualitative data analysis. All quotes have been translated freely from French to English. **Figure 2** offers visual synthesis of the themes associated with each of the three research questions.

### 4.2 Experience of MHPs learners in RC courses

In this section the experience of MHPs learners in RC courses will be described, according to three themes: (1) The format of the RC courses; (2) Types of knowledge shared; and (3) Expectations and recommendations.

#### 4.2.1 Format of the RC courses

All participants shared their perspectives on the format of the RC courses. Most participants expressed that there was added value in offering courses in an online format (n=11). Some participants valued this format because it was user-friendly and accessible, and reported on the dynamism of trainers, as demonstrated in the following quotes:

*I will underline the trainers’ dynamism, particularly on Zoom, where it is more challenging, as it can easily become monotonous. It seems they adeptly captured attention, benefiting from diverse facilitation techniques. Participant 9*

*The fact that it’s virtual, you know, makes it accessible. I don’t know if I would have … Would I have gone to Quebec for a full-day course? I don’t know, but having it virtually still allows us to … Participant 3*

Ten participants described the format of the courses as exchange-focused. These exchanges were characterized as warm, friendly or relaxed. The atmosphere was marked by active listening and gentleness. Clear emphasis was placed on the interactions between learners and trainers throughout the courses.

*It was really interactive, I think it’s truly something that doesn’t already exist. It’s a good interactive element, in the sense of the things that we did and thought about together. Participant 13*

Seven participants insisted on the distinct format of the RC courses compared to other recovery-oriented training programs. The course was described as novel, original, and surprising: the group composition and the coexistence of all types of knowledge within the group was often associated with this idea of novelty.

*And the fact that there were students, people with a mental health disorder, and practitioners from various fields [ … ], I wasn’t expecting that. My supervisor didn’t tell me about it. I thought it was a course for mental health practitioners. I was pleasantly surprised by this kind of change. Participant 11*

Five participants mentioned that the RC courses featured a straightforward format with short-term, achievable objectives. They noted that courses demanded a brief commitment and supported the attainment of goals.

*I would say it’s also an achievable course (laughs), in the sense that I can commit to it three times. And achievable in the sense that I also achieve something, like at the end of the course or during the course. It’s not just about medications or like … putting your whole life in order. It’s up to me to want to recover and then see that it’s possible, and that I can achieve it step by step, slowly, not all at once. Participant 1*

#### 4.2.2 Types of knowledge shared

Eight participants shared their experience by describing the types of knowledge they shared with others learners and trainers in the courses. Two major trends were observed: (1) Participants who were emphasizing clinical knowledge; and (2) Participants who were navigating through different types of knowledge.

Five participants endorsed a perspective where clinical knowledge was emphasized, manifested through their contributions to the group, their attitudes, their choice of words, or their interactions with other learners, as exemplified in this quote:

*When we were with service users or individuals who were not practitioners but rather peer support workers, it really brought another dimension. I think I quickly fell back into my role as a practitioner. I questioned the person, validated them a lot, and paraphrased. Participant 10*

Other participants (n=3) seemed to navigate from one type of knowledge to another. They reported adapting their discourses and the type of knowledge they wanted to put forward, depending on the nature of interactions, while focusing on the person-first, without considering titles and positions.

*I went there, I moved in the three spheres, taking the position of a lambda person [ … ], I didn’t position myself, I never mentioned what my job title was, or where I worked. Participant 7*

#### 4.2.3 Expectations and recommendations from the RC courses experience

All participants shared their initial expectations and some recommendations derived from their experience of the RC courses. Two major elements were identified: (1) Acquiring knowledge and tools for clinical practice; and (2) Enhancing the courses with a deeper integration of theoretical knowledge.

Participants mentioned some of their initial expectations, focusing on the acquisition of knowledge and tools that they could readily apply in their practice (n = 12). Among them, two participants particularly emphasized how they needed or wanted to gain tools that could help them in managing their own mental health. Some expressed that the tools that they could use in their clinical practice could also confer benefits at a personal level.

*My main objectives were to better understand the recovery process and its underlying principles. I also wanted to assess if I could integrate this into my current practice, and how to introduce it to my colleagues and the physicians I work with. It’s not a term commonly heard in my field. Participant 10*

Seven participants provided constructive feedback to improve the RC courses, suggesting a more prominent and comprehensive integration of theoretical knowledge. They described the theoretical foundations of the courses as basic; merely an overview, a refresher course, or a presentation of general definitions rather than a course that could advance the development of this type of knowledge. These participants were left unsatisfied.

*Coming from my clinical perspective, I have a thirst for knowledge, you know. So I would have liked to acquire more knowledge. I definitely felt unsatisfied. Participant 3*

### 4.3 Perceived benefits of the RC courses

This section will focus on the perceived benefits of the RC courses. Participants identified both clinical and personal benefits associated with their participation in a RC, indicating a connection between the two levels. These perceived benefits will be presented according to three themes: (1) Changes in clinical practice; (2) Awareness raising on clinical and personal issues; (3) Well-being and recovery.

#### 4.3.1 Changes in clinical practice

All participants reported changes in their clinical practice, following their experience in a RC course.

Ten participants mentioned that the course offered resources, information or tools on mental health, that could be applied or useful in practice, such as bibliographic references, visuals that represent the process of personal recovery, websites, and theoretical models. The participants reported that they could use these in their interactions with service users, their relatives or even with their colleagues in the workplace.

*The positive aspect has been the contribution it brought. Well, I would say it’s more related to knowledge directly linked to the subject. There are things that stood out to me, it helps me better understand resilience processes, truly work with it. You know, I’ve picked out bits that I’ve had said, ‘I have to share this with the relatives of service users I meet in my practice’. Participant 3*

Ten participants described how the courses had enhanced their understanding of the needs of service users and their relatives and challenges they faced. For them, the courses brought a deeper understanding of the lives of service users, an openness to different perspectives, and allowed to put words on the realities, experiences and feelings associated with living with a mental disorder.

*I can still imagine it, but when a mother comes to explain how she experiences it, and you know, not just one mother but two … And with two children who don’t experience it the same way … It helps me better understand the person, support them more effectively, and understand their needs as well. Participant 9*

Seven participants explained that the courses modified their assumptions, their perceptions and their biases regarding service users. In that sense, their experience in the RC allowed to build a renewed perception of what is their recovery or the journey toward recovery. This was facilitated by the exchanges between trainers and learners, which were filled with hope and inspiration.

*In the course of experiencing a particular situation or similar situations repeatedly, we are all susceptible to become disillusioned … There are clichés that we hear, like well, such and such person, we know psychosocial practitioner, will never get better, always messes up … As a psychosocial practitioner, our personal bias, at a certain point, leads you to try things with your client and sometimes, well, it feels like it’s going nowhere. I believe it can bring about a change in our perceptions fundamentally, so necessarily, we will also act in a different manner. Participant 1*

Seven participants indicated that the courses facilitated the integration of experiential knowledge, whether from their own experience or from the one of service users. For two participants who had experiential knowledge, the course allowed them to confirm that they were not ready to share personal experiences in practice. Participants described that they integrated more experiential knowledge into their clinical practice by allocating of more time for service users to express themselves, either individually or in group settings.

*I was facilitating a group [that the participant was leading at his workplace]. So it led me to reconsider things in a different way when it comes to group facilitation. The clinical impacts were precisely about taking the time, allowing people to speak, there was always this little reminder telling me, don’t forget this, it’s important. Participant 7*

Finally, seven participants explained that the courses enabled them to integrate recovery-oriented interventions more extensively into their clinical practice, such as promoting self-determination, encouraging peer support, adopting a strengths-based approach, or a systemic approach.

*I think that I am less … Well, I was going to say less prescriptive. For sure, I still need to be, but still a bit less. You know, as I was saying earlier, regarding medication. I think I am more nuanced, less … More into empowerment but also in explaining the reasons behind my recommendations. Participant 9*

#### 4.3.2 Awareness raising on professional and personal issues

Nine participants reported that the courses provoked clinical and personal reflections and raised awareness. Participants used verbs such as “reinforce,” “raise awareness,” “confirm,” and “remind” to describe the perceived benefits of the RC courses on this theme.

Nine participants indicated that their participation in RC reminded them of the importance of maintaining a humble attitude and adopting an egalitarian stance when interacting with service users.

*It prompted me to step back and gain perspective. We often emphasize that the service user is at the center of our practice in occupational therapy; the service user is at the center of our practice. However, it truly brought a sense of humility, encouraging me to reflect and consider the theoretical clinical knowledge versus experiential knowledge. Participant 10*

Seven participants shared that the courses raised their awareness of the universality of the experience of mental disorder: no one is immune from living them at some point in life. It is important to remain vigilant about one’s mental health, and the one’s relatives. To a certain extent, mental disorders are part of a normal life.

*On a personal level, it helped me to further normalize stress. Stress as a mental health practitioner can still be relatively taboo. Saying ‘I want to help 20, 30, 40 people a week, but I can’t manage my own stress myself ‘, is somewhat confronting. Sometimes, just talking about it, normalizing that everyone experiences it, reminds you that just because you have a specific job, you’re not immune to mental health disorder, stress, or depression. Participant 8*

Four participants indicated that the RC encouraged reflexivity on previous clinical situations encountered.

*Continuing education should encompass experiential elements; it should include a variety of perspectives, different ways of seeing things. This allows individuals, through introspection, to question themselves, ponder whether they are doing things well, and understand why someone else might approach things differently. It’s something we won’t have if we only focus on theories without incorporating our own ways of doing things, without adding our life experiences. Participant 8*

For some participants (n=2), this reflection focused mainly on the role of change agent and a desire to mobilize to have a positive impact on the delivery of mental health services.

#### 4.3.3 Well-being and recovery of participants

Six participants reported that their participation in the RC had an impact on their own well-being or their recovery journey. These participants explained that it allowed them to express themselves, to vent, and be truly themselves. They expressed that improving their own well-being enhanced their availability and their effectiveness in practice.

*I brought out experiences that I think I had never talked to anyone about, and having this space to do it, yeah, it was quite nice. It felt good to share what I shared, and it felt good to be heard as well. To see that, well, there were others who had experienced similar or slightly different things. Participant 7*

Four participants also mentioned that the courses offered practical tools for maintaining their own mental well-being and seeking help when needed.

*All the tools that were mentioned to express one’s identity, activate one’s body, mind, and senses, create connections with others, contribute to the community, to society. So, it’s everything that I keep in mind, something I can refer to. I try to put this into practice in my own life, and I can also assist the service users with it. Participant 12*

Finally, three participants mentioned that the courses facilitated improved self-awareness about their interests, strengths, and goals. Some reported progress in their recovery journey.

*I was listening to the trainer’s talk, and he’s like relaunching a career. It gave me the desire to be a nurse again, and to live with my limitations, and to accept my limitations. And to stop making society’s limits my own. You have to listen to yourself, and that there’s no failure, there’s no failure in resigning from that system. Participant 5*

### 4.4 Perceived key ingredients of RC

This section will describe what the participants identified as key and active ingredients of a RC. Four main elements were brought up by participants: (1) The group composition; (2) The interactions within the group; (3) The complementarity of the different types of knowledge; and (4) The pedagogical design and learning activities.

#### 4.4.1 Group composition

Most participants (n=13) observed the diversity of learners within the group. However, two participants noted an imbalance in their group, noting that there were many OT students and not enough learners with experiential knowledge. The similarities and differences observed primarily relate to the level of education, the diversity in the types of knowledge present, the and the job or workplace, the gender, and the age of learners.

*There was diversity among us. Some were mental health practitioners, some had just finished their studies in the health domain, I think. And there were peer support workers. I think I was the only nurse. There was the unit leader, so there was a manager. Yeah, it was from all walks of life, all ages too. Participant 5*

Six participants also indicated that this diversity of learners enriched the course, adding sources of information, different perspectives or ways of thinking.

*What I found truly enriching were the stories shared by individuals, offering diverse perspectives, —from a service user, a community organization, or within the healthcare system itself. You know, the health system, I hear about it, I know it more. Honestly, I believe the course would have been less interesting and less enriching if it had solely involved practitioners from healthcare institutions. Participant 9*

#### 4.4.2 Interactions within the group

Most participants described the interactions between trainers and learners or among the group as a distinctive element of RC. Twelve of them noted that the relationships were marked by an egalitarian stance and inclusion, where no hierarchy was observed. Learners and trainers were also equal footing.

*I didn’t sense any condescending individuals; I didn’t feel anyone discussing their experience with a certain superiority or judgment. I think it was truly egalitarian. Participant 8*

Eleven participants also mentioned the formation of varying degrees of interpersonal connection between learners throughout the sessions. These connections were described as a bond, a union, a sense of familiarity, or “shared insights” among learners. Collaborative work in sub-groups nurtured these connections.

*A really nice bond was forming and persisted, even during the breaks. It remained very personalized, and there was like a little something special, a small bond that had been created during the sub-group, and that persisted. Participant 10*

#### 4.4.3 Complementarity of the different types of knowledge

Twelve participants described the complementarity of different types of expertise and knowledge held by learners and trainers. They insisted on how the RC values the uniqueness, but also the differences amongst theoretical, clinical, and experiential knowledge, treating them equally. This fostered for participants a more nuanced and comprehensive perspective.

*We are all different, we are all unique, we all have knowledge, we all have a background, and no matter where we come from, whoever we are, we all have something to gain from each other’s experiences. There is no one better than the other, so I find it super interesting and rich to learn from everyone, actually. Participant 7*

Participants also mentioned the importance of experiential knowledge within the courses; nine participants described this being more authentic in RC than in other training programs. They indicated that experiential knowledge holds more meaning, evokes feelings and experiences, and enriches the courses.

*There were a few people who had really experienced moments of depression or diagnosed anxiety. So, it put a bit of words, feelings on how one feels when in that situation, experiencing that. I found it interesting to have, to put emotions, to put a perception of how the person feels, what worked, what didn’t work? I found it to be incredibly enriching, to be able to, not to put oneself in the shoes of, but to understand a perspective that is sometimes difficult to understand. Participant 7*

#### 4.4.4 Pedagogical design and learning activities

All participants identified features of the pedagogical design or the learning activities as a central element of a RC.

Most participants (n=13) cited the manifestation of many humanistic values that influenced the courses’ climate, that were demonstrated by both trainers and learners. These values encompassed respect and courtesy, openness, non-judgment, collaboration, compassion, kindness, authenticity, acceptance, and empathy. Such a climate nurtured confidence among learners, making them feeling welcomed and free to speak up and to participate in the proposed learning activities.

*Co-learning means openness, making room for mistakes, embracing compassion, maintaining an open mind, and acknowledging universality. I believe that everything is received with respect, whether it be theoretical knowledge or experiential knowledge. Participant 4*

Ten participants also indicated that they appreciated the pedagogical design of the RC. More specifically, the structure of the courses, which involves a variety of activities, made the time pass quickly. They acknowledged that this type of design requires from learners to adopt an active and collaborative role.

*I would really describe it as a type of collaborative learning. It involves making sense from multiple perspectives, bringing them together, and successfully advancing the process of reflection and reasoning. I truly believe that there is a co-construction happening. Participant 10*

Finally, ten participants explained that course design promoted assimilation, retention, and application of knowledge. Sharing drawn from experiential knowledge have particularly contributed to the retention of learning. One participant emphasized the role of feelings arising from experiential knowledge in this knowledge transfer process.

*If it’s someone who is an expert explaining things, and when I say an expert, it’s not just because they have theoretical knowledge but because they have the knowledge of having lived it and gone through it, well, that makes more sense because it’s a testimony of life, an experiential testimony. Therefore, affective memory comes to aid cognitive memory. Participant 7*

## 5 Discussion

The aim of this article was to describe the experience of MHPs learners, including OTs, in RC courses and the perceived benefits on their clinical practice. Participants described the format of the courses as exchange-focused, distinct, and straightforward. Offering courses in an online format added value to the experience, including making it more accessible. The participants also confirmed that co-learning in RC courses takes place within relationships marked by diversity, equality, and complementary knowledge.

Participants noted some benefits in their practice, such as an improved understanding of the needs and challenges faced by service users and their relatives, resulting in shifts in their perceptions and biases. RC courses offer mental health resources, information or tools on mental health, that can be applied or used in practice. They also support the use of recovery-oriented interventions with service users and their relatives or colleagues in the workplace. This is promising, given the limited evidence on the effectiveness of other recovery-oriented training programs on practice, such as the online webinar developed by the Substance Abuse and Mental Health Services Administration for acute care settings (69).

These findings align with previous RC studies. Participants recognized the distinct format and key and active ingredients of RC courses (70–73). They, like other learners, have acquired new tools beneficial for both professional and personal levels (59, 74). RC courses enhance understanding, empathy, and hope towards service users and facilitate the adoption of recovery-oriented interventions (58, 59). The findings support previous research indicating that RC courses underscore the importance of power equality within therapeutic relationships, promoting increased parity (58, 59, 74, 75). Interactions among diverse learners and trainers during RC courses fostered awareness of the universality and normalization of mental disorders (76).

These results also suggest that RC courses may contribute to the clinical reasoning of MHPs. According to OT literature, clinical reasoning refers to the cognitive processes practitioners employ during the planning, execution, and evaluation of their practice (77–80). Various types of reasoning have identified, including procedural, interactive, conditional, narrative, abject, pragmatic, generalization, scientific, ethical, and diagnostic reasoning (79–81). Interactive reasoning involves understanding the services users as individuals, including their perceptions of the events that prompted them to receive health services (77–80). Participation in RC courses enhanced participants’ comprehension of the service users’ needs and challenges. Procedural reasoning entails considering the disease or disability and selecting appropriate treatment activities (procedures) to improve the person’s functional performance (78–80). Following the completion of the RC courses, participants reported the adoption of a range of recovery-oriented interventions. These included the promotion of self-determination, the encouragement of peer support, and the implementation of strengths-based and systemic approaches. Finally, MHPs use narratives (storytelling and story making) to convey and discuss therapy events and activities with services users (77–80). Recovery narratives can facilitate a dialogue between MHPs and service users (82, 83). RC courses support the integration of experiential knowledge into clinical practice, encouraging expression through increased individual and group interaction.

The results of this study, along with the RC model literature, invite researchers and practitioners to reflect upon and to explore how RC courses can serve as a transformative tool within the healthcare systems. To understand what can contribute to this transformation, the educational approach used in RC courses seems to be relevant (71, 76). McGregor and colleagues (71) identified a connection between the theory of situated learning (84) and the elements of active learning, co-construction, and “making meaning” together experienced in RC courses. This observation resonates with Illeris’ theory of transformative learning (85, 86), encompassing cognitive, emotional, and social dimensions, and its relevance to RC educational approaches. The active engagement of learners and trainers in a co-constructed and egalitarian learning process where theory (cognitive aspect), experiential narratives (emotional aspect) and exchanges (social aspect) enable the creation of an integrated understanding of the issues, which may explain what makes the RC educational approach effective (48). Participants reported that experiential narratives facilitated the assimilation, retention, and application of knowledge. They learned in a different way than they were used to in other training courses. This may be one of the ingredients that enabled the adoption of recovery-oriented practice, along with other well-established ones such as organizational commitment (16, 42–44, 87).

For OTs, the RC model offers a pathway to move from a recovery philosophy to a recovery-oriented practice, benefiting both themselves and the service users and relatives they support. Through the use of this model, OTs may have the opportunity to foster collective intelligence aimed at effectively addressing mental health needs within communities. This transformative potential suggests that OTs could play a significant role in advancing recovery-oriented practice.

### 5.1 Study limitations and future perspectives

In spite of the use of a purposive sampling strategy to recruit participants, this research took place in a specific cultural context, which may affect the transferability of the results. It will be important to continue evaluating the experience and perceived benefits of MHPs, moving towards more scientific robust designs. Another limitation of the study is the lack of an objective description of the use of recovery-oriented interventions by MHPs before and after participation in RC courses. Therefore, a self-assessment tool for MHPs focusing on recovery-oriented competences and interventions is currently under development (88). We also noted that several participants of our study reported already having a recovery-oriented practice, with RC courses serving to confirm or remind them of the importance of certain attitudes, behaviors, and interventions aligning with the recovery paradigm. This highlights the need to objectively differentiate the level of “expertise” in future participant samples. For example, a study could be conducted with OT students, novice OTs, and senior OTs to examine whether the perceived benefits of RC courses differ according to clinical experience and their exposure to recovery-oriented practice.

## 6 Conclusion

This study highlighted RCs’ role in enriching MHPs professionally and personally. RC curriculum and courses drive changes in practice regarding mental health resources, attitudes towards service users, understanding of their challenges, and interventions. By fostering an inclusive learning environment valuing diverse knowledge, RCs help MHPs reflect on practice and improve clinical reasoning. This study advances understanding of a promising, accessible training program for adopting a recovery-oriented practice amid a paradigm shift among MHPs and OTs.

## Data Availability

The original contributions presented in the study are included in the article/supplementary material. Further inquiries can be directed to the corresponding author.
